# Growth Through Education: The Narratives of Older Adults

**DOI:** 10.3389/fsoc.2019.00011

**Published:** 2019-03-04

**Authors:** Cecilia Bjursell

**Affiliations:** Encell—National Centre for Lifelong Learning, School of Education and Communication, Jönköping University, Jönköping, Sweden

**Keywords:** narrative gerontology, narrative, story, older adults, older adults' learning, the aging process, Senior University, university studies

## Abstract

The focus of Narrative Gerontology is placed on stories about the aging process. In the present paper, the learning of older adults in a Senior University context is captured by means of stories written by the participants themselves. The examination of older adults' stories, as they look back on life or any narrative that connected to a specific area of life, can contribute to our understanding of growth later in life. The aim of the study is to examine how growth manifests itself later in life. Participants at Senior University were asked to share their experiences of education later in life. Participation was voluntary and the identity of each participant was kept anonymous for the purpose of the research project. Fifty-three stories written by Senior University participants (*n* = 38 women and 15 men) were analyzed according to: (i) an inductive analysis of the stories that resulted in a description of the main topics addressed in the stories, and (ii) a deductive analysis that invoked a theoretical framework concerning the existential aspects of older adults' learning, including “corporeality,” “relationality,” “spatiality,” “temporality,” and “materiality.” The two analyses were compared, and it was noted that “relationality” and “spatiality” corresponded to the educational experiences in the stories. “Relationality” was observed to be concerned with the social dimensions of life; but in the context of Senior University, “relationality” was strongly intertwined with the learning process. “Spatiality” addressed how older adults relate to physical- and mental space. Participation at Senior University entailed an expansion of both physical- and mental space for the participants. A number of tensions were identified in the stories. One the one hand, the stories can be interpreted as illustrations of moving forward and embracing continued growth and development. On the other hand, the stories can be interpreted as illustrations of resistance toward aging and decline. Since life is complex and contradictory, multiple, and even contradictory plots, co-exist in life stories.

## Introduction

The story of old age used to be characterized by themes of decline—resulting in the end of the story and the end of a person's life. However, since the proportion of older people is on the increase in many countries, we observe many people who have reinvented their lives, even in the later stages of life: “I know a woman who found a new love and got married when she was 80 years of age” or “I knew a man who, at 96, said that you have to keep moving forward.” Anecdotal, perhaps, but these stories resonate with a phenomenon that we can currently see in society; namely that “old age,” as a stage in life, is being extended. People are spending this stage of life in different ways. This new situation calls for alternative frameworks for the conceptualization of “old age,” and in response to this call, narrative gerontology is introduced in this paper so as to provide new perspectives on learning later in life. The aim is to explore and interrogate how growth manifests itself later in life and ask: *What do stories about participation in education provided by older adults reveal about the aging process?*

## Narrative Gerontology

Narrative Gerontology is a research approach that embraces storied dimensions in studies of aging. The metaphor, “life as story,” can be exploited so as to gain insights into the aging process itself, as well as how this process can be studied (Ruth and Kenyon, 1996; Kenyon and Randall, 1999). The life story, compared to fiction, does not necessarily follow a traditional dramatic structure. Although a life story can be framed within a grand narrative, it is more common that life stories are constituted of fragments and parts of memories. The presentation of a person's life by means of a narrative includes events of that person's “raw existence,” which are embedded in contexts and connected to the individual's perception of identity (Randall and Kenyon, 2004). How we tell stories about our lives has impact on what we learn, and, in turn, what we learn influences how we live our lives. “But the relationship between life, self, story, and learning is a complicated one.” (Goodson et al., 2010, p. 2).

Recognizing the transformative effects of stories, means that a narrative can be thus understood as a reflection of an internal view of development and aging. However, the story is also involved in the process itself. The examination of different narratives, for example, the narratives connected to work, family, religion, or the self, can provide a new understanding of the aging process (Kenyon and Randall, 1999). To narrate one's life story, or just a part of one's life story, is to engage in a process of becoming. To be encouraged to tell a story, and to move beyond the initial “this is the story-of-my-life,” holds the potential to strengthen an individual's resilience (Randall et al., 2015). It has been suggested that a person's willingness to share their life story later in life is indicative of their desire to live (Bruner, 1999). Independent of whether an older adult tells a story about what was or what is to become, the very act of creating a life story is indirectly saying that there is a life worth living. A life story emerges in the interplay between internal representations and the individual's social context (Kenyon and Randall, 1999) and is based on memory and an intention to re-present the past (Randall and Kenyon, 2004).

A story, like learning, is often triggered by an imbalance or tension of some sort (Bruner, 1999; Jarvis, 2004) and this imbalance provides the impetus for movement and potential transformation for the individual. According to the theory of gerotranscendence, the aging individual moves through a series of changes that imply redefinition of the self, as well as the individual's relationships to others. Gerotranscendence shifts the aging individual's perspective from a materialistic and rationalistic perspective to a metaperspective. This metaperspective is characterized by a more cosmic and transcendent view of the world. Tornstam (2011) claims an understanding of personal development later in life that is based on three dimensions of maturation: (i) the cosmic dimension, (ii) the self, and (iii) social and personal relationships. In this theory, the cosmic dimension addresses shifts in broad, existential perspectives where individuals connect their perceptions of the past, the present, and the future. The dimension of the self addresses how individuals view themselves and their experiences. The social and personal relationship dimension addresses how relationships with others are experienced and contextualized. The inclusion of existential aspects in studies of narratives about education can be worth pursuing because this approach is sensitive to the notions of “growth through education” and “growth through aging.” In one study of older adults' learning experiences, five existential aspects were identified (Narushima et al., 2018). These five existential aspects are:

1. *Corporeality*. Assurance for the dissonant body and mind. Learning as a means of dealing with the changing body.
2. *Relationality*. A circle of camaraderie. Creating social relationships and networks.
3. *Spatiality*. A balance between physical and mental spaces. Narrowing or expanding physical and mental spaces.
4. *Temporality*. Integration of the past, present, and future. Themes involving temporality are “the past” (time directed inward), “the present” (time is free to fill), and “the future” (time left to grow).
5. *Materiality*. Beyond knowledge and skills, “things,” tangible and intangible materiality, as extensions of our bodies and mind.

In the interrogation of the process of “becoming” in later life, narrative gerontology provides us with a useful framework which we can employ to understand gerotranscendence through the close examination older adults' stories. The analysis of these stories which constitute the empirical material in the present study was performed in two steps. First an inductive reading of the stories was performed, and then the stories were analyzed in the light of the five existential aspects suggested by Narushima et al. (2018) above. This approach allowed the researcher to connect the participants' voices (as present in the stories that they provided) to a theoretical framework that can be invoked in explaining certain existential aspects of gerotranscendence.

## Methods and Setting

The changing demographics of many countries reveal that their respective populations are expected to live long and relatively healthy lives after retirement. This is a new situation that needs to be examined closely because of the consequences it holds for these population groups. The role of education and learning, both in late working life and during retirement, is one area where we need more knowledge about the new situation.

### Studying Learning That Takes Place Later in Life

In their review of how older adult learners are portrayed in the literature, Chen et al. (2008) criticize the normative descriptions found there, where older adults are presented as “able-bodied” and “free from cognitive decline.” This portrayal of older adult learners “paints a picture of healthy, active retirees engaged in formal learning at universities and in Elderhostel, second careers, international travel, and so on.” (Chen et al., 2008, p. 10–11) Today, researchers now address the diversity and variety that exists in the population group; “older adults.” For example, Golding's (2015) research on the Men's Shed movement includes learning among older, poorly-educated men. Other studies have taken particular note of socio-demographic differences; including gender, age, and educational level (see Bjursell et al., 2017). The current paper does, however, address a somewhat privileged group; namely, older adults who are active and are able to retire comfortably. In many instances, this group represents white middle class retirees in Sweden. This group of people is of interest because: (i) they are used to participating in formal educational activities and, as a consequence of this experience, might hold different expectations when compared with people who have not had such experience, or perhaps they are unused to or have had a negative experience of previous formal education activities, and (ii) this group of people provide us with the opportunity to examine any connections that can be made between social class and old age, as we explore social change throughout life. The inclusion of ‘social class’ as a dimension is necessary if one wishes to understand the heterogeneity displayed by the group older adults, as well as the changes that are triggered by age (Formosa and Higgs, 2015; Higgs and Formosa, 2015). The examination of how older people view themselves in terms of “social class” may provide us with insight into what constitutes sources of “identity” and “changes in identity” later in life (Hyde and Jones, 2015). As suggested in the theoretical framework used in this paper, narratives provide us with access to sense-making processes, and therefore, participants at Senior University were asked to share their stories about participation in education.

### Stories About Participation at Senior University

Senior University represents the University of the Third Age movement in Sweden (U3A). This social movement consist of a number of non-profit, volunteer associations which offer courses and activities to their members. In Sweden, there are currently 34 Senior Universities, with an enrolment of 25,000 members (participants) in total. Senior Universities are organized as associations which are formally linked to the Swedish Folkuniversitet system, one of 10 educational associations that exist in the Swedish folkbildning system (a general-level education system for adults). Senior Universities are historically linked to the higher education system, but also in the sense that the content of the activities that take place under the auspices of Senior University are of a somewhat high academic level. Consequently, Senior University in Sweden resonates more with the Francophone U3A model than with the Anglophone model (see Formosa, 2014).

The empirical material that was used in this paper is based on Senior University participants' stories about their experiences of learning later in life. Part of this study will be presented in an forthcoming book chapter (Bjursell, 2019), but note that the empirical material that is presented in this paper has not been discussed earlier. Participants at Senior University were contacted by a letter which was sent to the governing board of each of the 34 Senior Universities. Most boards responded by stating that they did not wish to forward the letter to their participants; but 5 boards did forward the letter. In the letter, the researcher asked the participants to share their stories about participating in education later in life. The letter stated that any forthcoming materials were to be used for research purposes, participation was voluntary, and, if they chose to submit their story, their identity would be kept anonymous. Fifty-three letters with personal stories about their experience of education in later life were received. These letters contained a range of types of responses; from a few short comments to one response of five pages in length. The 53 letters were written by 38 women and 15 men. The excerpts from the letters that are found in the Results section below are anonymous, but the gender of the participant is shared (indicated by “f” for female participants, and “m” for male participants) and each letter was assigned with a unique number so as to indicate to the reader when multiple excerpts or quotations are made from the same letter. The majority of the respondents who shared their thoughts and experiences were between 70 and 80 years old. The oldest respondent was 93 years of age. It should be pointed out that the collection of narratives and reflections that was shared with the author does not constitute a systematic investigation, but, rather, it establishes an exploratory effort to come to an understanding of why one might choose to participate in the Senior University movement. The stories are not presented as single stories in the result section. Instead, they are grouped thematically so as to illustrate a collective understanding of what participation at Senior University entails. The Results section remains close to the original narratives and many quotes are included so as to allow the reader to take note of the participants' own words and thoughts. As such, they are to be understood as narratives, rather than as a life story. Notwithstanding this caveat, the reader should note that they are narratives that represent an important part of each participant's life story: the part that education plays in the participants' lives.

This study was carried out in accordance with the recommendations of Good Research Practice provided by the Swedish Research Council. All of the participants in this study provided informed consent, in writing, in accordance with the Helsinki Declaration.

### Strengths and Limitations

One strength of the narrative approach is that it allows for the analysis of the complexity and contradictions that occur in life. The use of the participants' written stories, rather than oral interviews, is another strength of the study, since this approach enables the participants to construct their story in the way that they prefer. The analysis of the stories, however, was performed by the researcher; something of both a strength and a limitation. This method is a strength since the researcher is not emotionally invested in, or identifies with, the stories. It is a limitation since there is always the danger of presenting a mis-interpretation of the story, which could have been avoided if the participants were involved in the analysis. A suggestion for future studies is to involve the participants in the analysis in a way that combines distance (objectivity) and closeness (subjectivity). Another limitation of the study concerns the group of participants. The participants are from a single country (Sweden), they chose to participate themselves, and they attend Senior University. These factors usually entails that the participants are middle-class professionals. Although the results of the study may be generalized to describing personal growth later in life, the results of this paper should be understood as being representative for only the group under investigation.

## Results From the Inductive Analysis: Voices About Older Adults' Learning

When one asks participants at Senior University why they are involved in the association's activities, two main points are raised: (i) participants wish to further their education by acquiring new knowledge, and (ii) they wish to be part of a social community with other, like-minded people. In some cases, the social aspect was a requirement for learning, since it provided a secure forum where the participants felt comfortable enough with each other to engage in dialogue.

### Studies Aimed at *Bildung*

In addition to being a forum for an active life in general, the participants reported that Senior University provided them with opportunities for further study and continual education. Retirement can be a period in one's life when one can fulfill a life-long dream of continued studies or further reading on a subject which does not necessarily have a practical application. One female respondent wrote that she “greedily enrolled in a large number of courses, right from the start”:

For the first term, I enrolled in ten courses, a beginners course in Italian, water color painting, Art History, and many others. But my main interest was, and remains, in the Writer's Workshop. I love to write, but like so many other people, my work was left at the back of the drawer. Already at my first Writer's Workshop, I dreamt of being the leader of a writer's circle—and, after some time, I became one. (2018, p. f19)

It is not uncommon for women to suppress their desire for further studies during their careers and while they are raising their families, but when they retire, new opportunities for self-actualization are opened up to them:

So you ask, why should one wait? Duty, duty. Marking assignments on the weekends. Any free time was spent helping my dyslexic son and maintaining contact with my friends. Doing exercise. And, of course, the household chores. […] When I retired, I thought: Eventually! Now I can study whatever I like. I can write and study interesting subjects. After my retirement, I thus studied at university, some courses at Linköping, Stockholm, and most recently at Gothenburg, since it was only there that one can read for an International Master of Art with an emphasis on the theory of art. Whilst employed as a high school teacher, I taught the history of culture and philosophy and I am interested in art, but I never read Art History at university before. (2018, p. f07)

Among the participants' narratives, there were also examples of individuals who wished to continue with their studies after retirement even though they had worked for a living and were engaged in further studies at the same time.

I had a full-time job, taught courses at night, and also attended courses, so it was a natural choice for me. (2018, p. f49)

There are several directions that the participants' learning took. Some participants wished to engage in advanced studies where they continued to study an area which they had already mastered. Others wished to learn about a subject which they had not had previous opportunity to study. One aspect of further study after retirement that was observable in the narratives was the appreciation of the fact that it is important to keep one's brain active, especially in old age. For some participants, the study circle's open format did not suit them and, consequently, they enrolled at university. Finally, there were many participants who viewed Senior University as a forum for further discussion in a number of *different* areas. This view was maintained because they wished to satisfy their sense of curiosity, and to deal with existential questions in open discourse. With respect to different approaches to studying, the participants' reports were divided across three general approaches: (i) further studies after retirement, (ii) moving from Senior University to university studies, and (iii) broadened enlightenment.

#### Further Studies After Retirement

One reason why an individual might choose to participate in an activity at Senior University was that doing so provides stimulation and a context for learning activities.

In connextion with my retirement, I really thought about what I was going to spend my time and energy with, but also [I thought] about a context which I could be part of. when my children were young, I took some night courses in French for several years, but I stopped going to these classes when I was accepted as a doctoral student and realized that I would not have time for both my language studies and my doctoral studies. But when I retired, the dream to continue my French studies reappeared. I chose Senior University because I thought that their courses were serious but without the demand of obtaining university credits. (2018, p. f31)

Many Senior University participants had previously achieved high levels of educational attainment and were used to being active in further education during their careers. In contrast to the professional development courses that they may have taken during their working careers, language studies was a popular area that participants engaged in.

When I retired, I thought that I had the time to improve my German, which I used to some degree previously, but by then had become a bit rusty. The course is nice and rewarding, which is why I enrolled on the English course, a language which I am quite good at. For the second course, I was not looking to improve my language skills that much, but we do have interesting discussions and read enjoyable literature. (2018, p. m52)

I am on a French course which is given once a week. On top of this, I spend some time doing homework, perhaps 3 to 5 hours per week, depending on the difficulty of the text and the grammar. I have also come into contact with L'Alliance Française via this course, an association which works to spread and support the French language and culture to anyone who is interested in this. This has provided me with an additional context where I can be part of with interesting talks and a pleasant fellowship. (2018, p. f31)

Learning a language through conversational courses seemed to be a popular activity amongst the participants and whilst some people attended these courses for their social aspects and to keep their brains active, there were also some attendees who were interested in the practical use of advanced language skills.

I would really like to keep my knowledge of English alive. I have a daughter in the USA and frequently travel there, so I enrolled and I have continued [with my English studies] for several years now. (2018, p. f23)

I am part of a group of people who are reading Modern English Literature at Senior University. We thus read newly published literature in English and discuss them in English in the group which meets every 3 weeks. Because I have read a great deal my whole life (I am 74 years old now), I consider it to be a natural and an important part of my life. Reading in English is not about learning about something new, but, rather, it is about trying to retain my previous knowledge. Because I travel a great deal, it is important for me to maintain my level of English as best as I can. (2018, p. f21)

By immersing oneself in an already well-known area, one may keep one's existing knowledge alive, but it is also a way of further developing one's knowledge. Another effect of this is the experience that doing so “gives you self-confidence and increased status.” (2018, p. f32) Connecting to the theory of gerotranscendence, studies at the Senior University could be interpreted both (i) as a way to self fulfillment, pursuing studies for their own sake, and (ii) as resistance to aging, since participation was also described as a way of upholding one's status and position in society. In the narratives, the study groups were described as including like-minded individuals.

The homogeneity [of the group] gives one a certain comfort, I think. In my French group, we talk in French about something, what we did during the week. For example, about a book one has read, a film one has watched. This reveals a great deal about oneself as a person, even one's social status! This might be an obstacle for someone who does not “belong to the group.” The same applies to a seminar group which I participate in, an art appreciation association, a type of study group. This group is also a group of “attuned” women, retirees with good education and, in many cases, with husbands who have done well for themselves. Culture vultures, you might say. (2018, p. f07)

The majority of the participants who chose to write about their participation at Senior University seem to be people with a large amount of cultural capital. It is possible that this is not representative of all Senior University participants, but within this group there seemed to be a further dimension of wanting to retain, or even increase, one's cultural capital. Situations can thus arise where the level of the courses and activities do not fulfill the participant's expectations. Some people who were not met with the challenge or level which they expected moved on to study at university instead.

#### From Senior University to University Studies

The courses at Senior University are provided by pensioners, for pensioners. Thus, the course lecturer and the participants of the study group decide on the content and level of the course together. Consequently, levels can vary a great deal. Amongst those who sent in their reflections over their participation in Senior University, some were of the opinion that Senior University did not offer a sufficiently advanced level of study.

I believe that Senior University, at least in my home town, is not aware that we are pensioners who are well-educated and, during our careers, received good further training which has lead to a passion for learning. (2018, p. f26)

Participants who possessed a university degree usually had higher expectations and consequently placed higher demands on the course leaders. This included how they presented the subject, if they could respond to the needs of the mature participants, and if they could evaluate and adjust the level of a course that was presented to a specific group. Participation in Senior University activities could also be a gateway to enrolling onto a course as a pensioner. Some people then discovered that they were searching for greater challenges and then moved on to university studies.

I thought that once I had turned 65 I would lie in bed reading books and eating chocolate. But then I enrolled at Senior University and took some courses. After some years I realized that these courses were at a level that was too low for me. I needed more to keep my brain active. So then I enrolled on a 15 point credit course in economics. It was great fun. I was able to learn so much and just enjoy myself. I was worried that the young students might not accept me but there has never been a problem. That is how it is. I feel at home there. (2018, p. f26)

I have been a pensioner for almost five years. Soon after retirement, I started with a French course at Senior University, the most advanced level. I have continued with it since then, but I don't attend any more Senior University courses. If there was a similar course for German which did not clash with the French schedule, I am sure I would have attended that course too. Instead, I enrolled on a quarter-paced course in German at university. (2018, p. f42)

I have discovered another way to satisfy [my desire for] knowledge and learning, namely individual university courses. […] These are interesting but demanding courses, which are taken with students between 20 and 30 years of age, and a few older students. This allows for exciting perspectives and exchanges by socializing across the different generations, something which I find appealing. (2018, p. m16)

The degree of formality and the educational level is different when one compares Senior University with traditional university courses, but if the individual has the prerequisite knowledge, interest, and enthusiasm, then university studies can be an alternative for people who are looking for a certain type of intellectual challenge. From the perspective of gerotranscendence, the descriptions in the narratives about going to a traditional university were similar and expressed a resistance to aging by the participant maintaining the same level intellectual level as before. Furthermore, university attendance also held a relational dimension for the older students, who were able to interact with likeminded peers and the younger generation. In other words, the growth process seemed to be the same across these two different institutions, although the level of studies was, of course, different.

#### Broadened Enlightenment

Whilst some individuals may have chosen to deepen their knowledge of a “school subject,” there were others who were searching for a broader understanding of life's various aspects. The mere act of studying had value in itself, besides the subject content that was studied.

Learning is part of the meaning of life. I think this is particularly relevant. I am curious, I like to feel competent in new areas; I like to share learning and knowledge with others. (2018, p. f15)

I believe that Senior University suit us who are always curious. Perhaps we have had an intellectual career or, in our old age, have discovered that there is so much around us which we wished we could know about and understand; languages, about other cultures, about art, about the environment. (2018, p. f19)

The study of culture (in its various manifestations) was popular and the pensioners took on the roles of both observer and practitioner. One person described that his participation on a course on jazz was a highlight, especially since the study circle was lead by Nisse Sandström, a prominent figure in Swedish jazz.

The jazz circle was in a class of its own. […] We attended two smash hits at the Nalen [concert hall] in Stockholm where we got to listen to some noble jazz including Nisse Sandström who played solo and we got to dance to the tones of a big band. (2018, p. m30)

The same person described himself as a “technology nerd” and was especially interested in study visits at industrial plants and buildings, but he also thought that visits to senior cinema were rewarding. Some of the women described themselves as “culture vultures,” a term which is sometimes used to refer to older women because they tend to be a dominant force among consumers of culture.

I am quite the culture vulture who attends concerts and the theater. I addition to what you can learn as an older person (as long as your mind can keep up) the social fellowship you have with others is also valuable. I have made new friends at my French studies at Senior University and I am happy about that! (2018, p. f42)

An interest in culture, as mentioned previously, can also include oneself being part of a creative process and meeting others from whom one can learn and be inspired by. This may be appealing to someone who does not desire to continue with learning in a more theoretical area.

I don't attend courses. This might be because I live outside town. It is too complicated to journey in to town. It has to be a very interesting thing if I am going to make the journey. I have no desire to participate in theoretical courses. I finished with that at school. I am a creative person and I keep myself busy at home with embroidery, sleeping, and the garden. If any courses that were interesting were offered in my village, then I would participate. I participate in Senior University's trips, group lunches, the cinema, and certain field trips. Then I get out and about and get inspiration. (2018, p. f48)

“Broadened enlightenment” may be the result of studying the content of a course, but it also may emerge when meeting other people where one might be challenged in various ways. The desire to know more and to reflect over one's thoughts together with others drives people to look for contexts where there is room for discussion.

We believe that many older people view Senior University as a particularly appealing opportunity to broaden one's knowledge and to discuss issues that are of concern to us. (2018, p. f53)

What makes the difference is, of course, the multifaceted range of experience which is present in groups of old people and which often gives rise to more existential questions being put on the agenda. (2018, p. m27)

Being willing to enter into a dialogue with other people who also possess a great deal of life experience provides the conditions where one can speak about things in a way which is perhaps more difficult with young people, who find themselves in a different stage of life. Conversations with others allows one to broaden one's horizons and one's sense of enlightenment about life in general; not just knowledge of specific facts. This could be an indication of a shift toward a more cosmic and transcendent view of the world, which is a sign of maturation as we age, according to the theory of gerotranscendence.

### The Fellowship Experienced During the Courses

As mentioned previously, Senior University provides participants with a forum for learning and for social fellowship. The participants reported that there was time for social interaction before, during, and after the activities that took place.

I plan to continue with my language courses, because I want to be more proficient, but also for social reasons. (2018, p. m52)

I attend senior cinema with my childhood friend. We meet up at least one hour before the viewing for a cup of coffee or lunch. Most of all, we get to talk with each other. At the cinema, I meet old friends and ex-work colleagues and we chat together. (2018, p. f08)

One of the participants reported that, when he moved to a new town as a pensioner, Senior University became an important channel for him in finding new social contacts. One person who is on the managing board of one Senior University claimed that many members go on trips and attend courses for reasons associated with social interaction. This was especially the case for people who live alone; the association is important for maintaining social contacts.

I am most interested in the lectures, trips, and field trips. You also get to meet very nice people and there is a good social spirit. All these things are very positive. (2018, p. f50)

The social dimension of participating in Senior University activities is not just about meeting other people and preventing social isolation. By keeping oneself up-to-date in different fields, one is also able to follow social developments and one can feel that one can still contribute to society as an active citizen.

It is both rewarding and stimulating to be able to be engaged in social debates using recent research and this entails that any sense of isolation is kept at bay, which is something that can easily happen if one isolates oneself [from society] after one retires. (2018, p. f05)

We thus note how intellectual and social stimulation can go hand-in-hand. Meeting together, around a common area of interest, can offer greater opportunities for learning since one is able to hear of other people's experiences and their knowledge; something which is considered positive.

I am happy that, as a pensioner, I had the opportunity to study and obtain knowledge in an area that has always interested me, but, because of different reasons, I was not able to pursue this earlier. Of course, it is always possible to read and study on your own, but it is more rewarding to do this in the company of other people, since we can share our experiences and knowledge with each other in a small group. (2018, p. f33)

The social fellowship that can be enjoyed within a study group is thus particularly important; it even contributes to the intellectual stimulus felt by the participants. Belonging to a group where the participants share certain characteristics was described to further the social interaction that took place.

So then I think that one should choose the association that fits in with your personality. “Birds of a feather” is an expression which I think applies to one's whole life. (2018, p. f05)

The participants knew each other; they seemed to keep track of each other. This differentiated this group from others. The older members were happy to talk with each other, they weren't so stressed out and neither were they inhibited. They didn't have to put on a [false] show. (2018, p. f07)

In the autumn of 2016, I started to learn Spanish together with 10 older ladies with various levels of [Spanish] proficiency. We were a nice group together, who accepted each other's different levels of knowledge, hearing problems, and so on. (2018, p. f51)

The similarities shared across the participants created a spirit of acceptance and security. Being part of an environment that was solely made up of older participants was said to support the interaction between the participants, since they shared the same pace of life and had empathy for each other concerning certain ailments, for example, being hard of hearing. The notion of “status” may also play a role in being able to identify with the others in the group; as a reflection of one's own identity. The search for a context or environment which is characterized by a spirit of acceptance or where one can maintain one's previous status in life and one's identity are manifestations of different aspects of the aging process. There are actually courses and lectures at Senior University which explicitly deal with the subject of old age and the aging process, but a number of the participants that sent in their stories reported on actively avoiding this issue.

I consistently refrain from attending any lecture about old age and all the terrible things that can arise. (2018, p. f37)

The avoidance of “old age” as a theme may well be a priority for certain individuals, but it may also be the realization of some form of resistance to being involuntarily assigned to this demographic category, because old age is stigmatized to some degree in Swedish society. A recurring question is what one should call older people. One participant put it thus:

*It should be noted that I don't like to be called “old”, like in this questionnaire. It's a form of age discrimination! In newspaper articles and in many other contexts, a large proportion of the population are bundled together and called “the elderly”. For example, I read in the newspaper “older woman robbed”—she was 58 years of age. At Senior University, physically- and mentally healthy and youthful people between 65 and 95 years of age get together. Save the term “the elderly” for questionnaires that concern care for the elderly and retirement homes, and so on. Why don't you use the word* *senior**? (2018, p. f03)*

There are others who do not like the term *senior*, and one respondent had a problem with the word *pensioner*. Irrespective of how one chooses to label groups of people and even individual people, we are faced with a situation in Sweden where the group, “older adults,” is a large and heterogeneous group of people. If we note that the lower age limit for Senior University is 55 years of age and that many people now live up to a 100 years of age, this entails that the participants at Senior University actually represent several generations. There is, however, one thing that most people in this group have in common—they prefer activities that are voluntary and flexible. In terms of their learning, they enjoy the study circle form of education because it is based on a dialogical approach were experience plays an important role. “Social- and personal relationships” is one of the three dimensions of the maturation process later in life (Tornstam, 2011), and to engage in dialogue is relevant to the process of gerotranscendence.

## Results From the Thematic Analysis: Existential Aspects of Education Later in Life

This section presents the results from the deductive analysis that invoked the theoretical framework concerning the existential aspects of older adults' learning: “corporeality,” “relationality,” “spatiality,” “temporality,” and “materiality.”

### Corporeality: Assurance for the Dissonant Body and Mind

*Assurance for the dissonant body and mind* refers to how participants talk about the bodily changes that occur in conjunction with the aging process and how they deal with these changes. Narushima et al. (2018) report on how participants were sometimes frustrated and anxious about their physical decline and the illnesses that they sometimes suffered from, but also how participants can overcome physical decline by using different strategies, such as forcing themselves to go to places even though, for example, their knees were painful. This has been described as “Mind over matter.” A difference between the present study and Narushima et al.'s study is that their study included participants who were vulnerable older adults. In the present study, the participants described themselves as “active” and “healthy.” The presentation of one's self as “active” and “healthy” is further supported by comments made by the participants that they do not belong to the category, “the old people.” For example, “It should be noted that I don't like to be called “old.”” To be called “old” was considered tantamount to discrimination by the same respondent; thereby implying that “being old” is derogatory. There is thus a silent resistance against being identified with “aging” and “physical decline,” which may also signal a resistance against gerotranscendence and positive aging. In some of the stories, physical decline is hinted at: “We were a nice group together, who accepted each other's different levels of knowledge, hearing problems, and so on.” The context of being together with older adults can thus provide a safe and comfortable environment where physical changes are recognized as something natural. An acceptance of the aging process is not a sufficient and necessary condition for gerotranscendence, however. Notwithstanding this, one physical issue that openly recurs in the narratives is the issue of keeping the brain active. The participants recognized that it is necessary to lead an active life, and in the context of education, there was a focus on retaining the same cognitive level or even interest in further developing one's cognitive capacity. Participation in educational activities can thus be understood as a means of dealing with aspects of corporeality.

### Relationality: A Circle of Camaraderie

*A circle of camaraderie* refers to how people create social relationships and networks. With increased age, there is an increased risk for the loss of social networks and a concomitant risk of social isolation. Narushima et al. (2018) describe how participants gained a sense of belonging to a community and built informal networks because of their participation in educational activities. This observation is congruent with what was found in the present study, where a number of participants remarked on the importance of the social dimension in connection with their studies at Senior University. In fact, for the participants included in this study, it was one of the two main reasons why they attend courses at Senior University. One difference, however, can be found in how Narushima's et al. (2018) participants spoke about “older people as role models” and “the important role of the instructor.” The narratives included in the present study, instead, contained stories about wanting to be the instructor or contained complaints about the instructors not offering sufficiently advanced levels of study. The social dimension found in the stories in the present paper included the community within Senior University and other communities, such as L'Alliance Française, and being a student at traditional universities and being an active citizen in society at large: “It is both rewarding and stimulating to be able to be engaged in social debates using recent research.” The stories also link the intellectual and the social dimensions, as can be seen in the report that: “the multifaceted range of experience which is present in groups of old people and which often gives rise to more existential questions being on the agenda.” The link between the intellectual and the social dimensions can, furthermore, be understood as representing gerotranscendence, since it enables the individual to move into a more universal perspective on life. Senior University thus provides a forum where learning and social fellowship are interlinked. Relationality is thus a properly integrated and prominent part of learning and education.

### Spatiality: A Balance Between Physical and Mental Spaces

*A balance between physical and mental spaces* refers to the participants' perceptions of narrowing or expanding physical and mental spaces. Narushima et al. (2018) found that, while the participants' physical space became narrower, their mental space expanded. In the stories from participants at Senior University, it was reported that their physical space as well as their mental space had expanded. Their physical space was broadened in terms of their course attendance, study visits, visits to museums, and trips to other countries for cultural exchange and experience. Only one of the 53 stories contradicted this general trend: “*I don't attend courses. This might be because I live outside town. It is too complicated to journey in to town*.” (2018, p. f48). The participants' stories indicate that their mental space was broadened, both intellectually and culturally. An intellectual broadening took place because the participants were challenged to develop their knowledge of a subject that was already familiar to the person or by taking on a new subject. Language studies were subjects that were referred to in this way in the stories. Cultural endeavors, on the other hand, entailed “performing” culture as well as “consuming” culture, but note that such endeavors also contained the notion of “a broadened enlightenment” through engaging in discussions about, for example, existential issues. This hints at the process of gerotranscendence described in the theoretical section. Spatiality, expanding one's physical and mental space, is a major theme in the stories about participation at Senior University.

### Temporality: Integration of the Past, Present, and Future

*Integration of the past, present, and future* refers to themes that involve and integrate the past, the present and the future. Narushima et al. (2018) describes how participants enjoyed the freedom of “now” as well as a shared willingness to keep growing and moving forward with time. Although they realized that the future was limited, participation in educational activities provided them with a structure in the present. The stories from Senior University shared these ideas. The participants reported that they could engage in whatever they want to and they had chosen education activities at Senior University to them with tools for growth and with a way of “keeping up with the times.” In terms of looking back on one's life, these stories were produced by women who claimed that they did not have the opportunity to study before, but now could realize their life dream: “*Duty, duty. Marking assignments on the weekends. Any free time was spent helping my son with dyslexia and maintaining contact with my friends. Doing exercise. And, of course, the household chores. […] When I retired, I thought: “Eventually! Now I can study whatever I like*.” (2018, p. f07). The stories about participating at Senior University also had a dimension of “keeping up with life, like before,” which is associated with the previously-mentioned claims concerning an active life and a stimulated brain. One notes a certain tension when one analyses the theme of “temporality” in the stories. On the one hand, the stories can be interpreted as “moving forward” and “embracing continued growth and development.” On the other hand, however, the stories can be interpreted as statements of “resistance against aging,” and “resistance against physical decline.” Because life is often complex and contradictory, I claim that both interpretations exist simultaneously.

### Materiality: Beyond Knowledge and Skills

*Beyond knowledge and skills* refers to tangible and intangible materiality, which is seen as extensions of our bodies and mind. As in Narushima et al. (2018), the stories from Senior University do not focus on tangible materiality. Instead, it is intangible materiality that is of interest to the participants. Their focus on the intangible may be a function of the context on which the narratives reported, namely education, or as an expression of the aging process. This issue should be discussed further in future studies. In the stories about participation at Senior University, there was less focus on “self-esteem” and “confidence” than that in Narushima et al. (2018), where these issues emerged as central issues. Instead, “social position,” and “the maintenance of the position the person held prior to retirement” were frequently described in the stories. Several stories emphasized the importance of remaining within a homogenous group of people who share a similar life situation: “*So then I think that one should choose the association that fits in with your personality. “Birds of a feather” is an expression which I think applies to one's whole life*.” (2018, p. f05). In some stories, the idea of enrolling at Senior University is dismissed because certain participants were of the opinion that the studies that were on offer to them were not intellectually challenging enough: “*[…] I realized that these courses were at a level that was too low for me. I needed more to keep my brain active. So then I enrolled on a 15 point credit course in economics*.” (2018,p. f26). In other stories, the level of cultural attainment and content is decisive for social positioning: “*For example, about a book one has read, a film one has watched. This reveals a great deal about oneself as a person, even one's social status!*” **(**2018, p. f07). Since attendance at Senior University has connotations with “higher education,” this implies that one holds a social position. This corresponds to the dimension of “self” in gerotranscendence and the process of reflecting on the differences between “identity,” “social position,” and “self.” It should, however, be emphasized that many stories describe an interest in continued growth, independent of the participant's social position. If education, and learning in general, represent intangible materiality as a process for growth later in life, Senior University could be said to represent a context where social positioning can take place as well. In other words, the process of learning (in a formal educational setting, or elsewhere) should be regarded as a process of growth, no matter at what stage in life a person finds herself.

## Comparing the Results of Deductive and Inductive Analyses

This paper began by introducing narrative gerontology as a theoretical framework within which one can conceptualize the personal growth that takes place later in life to explore the process of gerotranscendence. As suggested in the theoretical framework of this paper, the stories told by participants provided access to various sense-making processes that revealed different dimensions that are relevant to learning later in life. The group of older adults that shared their stories about their participation in educational activities at Senior University is a privileged group in many ways; including their educational background and social position. We also note that the individuals who submitted their stories for analysis were of good health (as far as the stories reveal). The examination of the participants' stories included two steps: an inductive analysis which resulted in a presentation of the material that was based on what was said in the stories and a deductive analysis of the stories that was based on the existential aspects presented by Narushima et al. (2018). The first analysis provided a rich understanding of the content of the stories. We noted two general arguments why a person might engage in studies later in life: (i) the participants wanted to acquire new knowledge and (ii) they valued being part of the social community that the Senior University offered. The second analysis treated five existential aspects: “corporeality,” “relationality,” “spatiality,” “temporality,” and “materiality.” When we compare both analyses, we note that the “social” dimension corresponded to the existential aspect of “relationality,” something which should be understood as an integrated and prominent part of learning and education, and to “spatiality,” suggesting that participation at Senior University is a means for the participants to expand their physical and mental space. In the inductive analysis, a tension was identified within the existential aspect of “temporality,” namely between maintaining the idea of being the same person, “as one had always been,” and engaging in continued growth and development. Based on the narratives, it is not immediately apparent whether “looking back” is a sign of resistance (“staying the same”) or whether it is a sign of acceptance (“embracing the “now” as a positive development of the past”). This tension occurs in the other existential aspects as well.

## Conclusion: Maturation Through Education in Later Life

The major insight that is offered by the present study is that *multiple and contradictory plots co-exist in stories about participating in education later in life*. This corresponds to the notion that life itself can be complex and contradictory. Different plots in these participants' life stories are embraced, suggesting that different plots exist simultaneously and represent the aging process as a combination of “holding back” and “moving forward.” When the movement of personal development is perceived as positive, this is often referred to as *maturation*. The theory of gerotranscendence explains this movement. Tornstam (2011) identified three dimensions of maturation that takes place later in life: the “cosmic” dimension, the “self,” and “social and personal relationships.” In the narratives received from the participants at Senior University, development of the “self” and of the “social and personal relationships” was a recurrent theme, thus indicating that education and learning can play a valuable role in gerotranscendence. The “cosmic” dimension was not immediately obvious in the stories; which does not mean that it is absent from the narratives. It may be the case that the participants were more at ease talking about the “self” and “social and personal relationships” compared to the “cosmic” dimension of maturation. In fact, the “cosmic” dimension was touched upon with mention of “broadened enlightenment” and the notions of “being an active citizen” and “participating in society.” But most narratives were about development in terms of “self-realization” and individual goals. Is it that the participants at Senior University, a group consisting of older adults who are relatively healthy, active and well-educated, find themselves at the early stages of the gerotranscendence process? They may even be in denial of the aging process? Or it may well be the case that Senior University constitutes a forum for education were the focus is placed on the individual' and the social dimensions, instead of on the “cosmic” dimension? The answer to these and many other questions, remain for future research to address.

## Author Contributions

The author confirms being the sole contributor of this work and has approved it for publication.

### Conflict of Interest Statement

The author declares that the research was conducted in the absence of any commercial or financial relationships that could be construed as a potential conflict of interest.
